# Swine farm groundwater is a hidden hotspot for antibiotic-resistant pathogenic *Acinetobacter*

**DOI:** 10.1038/s43705-023-00240-w

**Published:** 2023-04-20

**Authors:** Fang-Zhou Gao, Liang-Ying He, Xin Chen, Jing-Liang Chen, Xinzhu Yi, Lu-Xi He, Xin-Yi Huang, Zi-Yin Chen, Hong Bai, Min Zhang, You-Sheng Liu, Guang-Guo Ying

**Affiliations:** 1 SCNU Environmental Research Institute, Guangdong Provincial Key Laboratory of Chemical Pollution and Environmental Safety & MOE Key Laboratory of Theoretical Chemistry of Environment, South China Normal University, Guangzhou, 510006 PR China; 2 School of Environment, South China Normal University, University Town, Guangzhou, 510006 PR China; 3 Institute of Ecological Science, Guangzhou Key Laboratory of Subtropical Biodiversity and Biomonitoring, Guangdong Provincial Key Laboratory of Biotechnology for Plant Development, School of Life Sciences, South China Normal University, Guangzhou, 510631 PR China; 4 MRC-University of Glasgow Centre for Virus Research, 464 Bearsden Road, Glasgow, G61 1QH UK

**Keywords:** Environmental sciences, Water microbiology

## Abstract

*Acinetobacter* is present in the livestock environment, but little is known about their antibiotic resistance and pathogenic species in the farm groundwater. Here we investigated antibiotic resistance of *Acinetobacter* in the swine farm groundwater (JZPG) and residential groundwater (JZG) of a swine farming village, in comparison to a nearby (3.5 km) non-farming village (WTG) using metagenomic and culture-based approaches. Results showed that the abundance of antibiotic resistome in some JZG and all JZPG (~3.4 copies/16S rRNA gene) was higher than that in WTG (~0.7 copies/16S rRNA gene), indicating the influence of farming activities on both groundwater types. *Acinetobacter* accounted for ~95.7% of the bacteria in JZG and JZPG, but only ~8.0% in WTG. They were potential hosts of ~95.6% of the resistome in farm affected groundwater, which includes 99 ARG subtypes against 23 antibiotic classes. These ARGs were associated with diverse intrinsic and acquired resistance mechanisms, and the predominant ARGs were tetracyclines and fluoroquinolones resistance genes. Metagenomic binning analysis elucidated that non-baumannii *Acinetobacter* including *A*. *oleivorans*, *A*. *beijerinckii*, *A*. *seifertii*, *A*. *bereziniae* and *A*. *modestus* might pose environmental risks because of multidrug resistance, pathogenicity and massive existence in the groundwater. Antibiotic susceptibility tests showed that the isolated strains were resistant to multiple antibiotics including sulfamethoxazole (resistance ratio: 96.2%), levofloxacin (42.5%), gatifloxacin (39.0%), ciprofloxacin (32.6%), tetracycline (32.0%), doxycycline (29.0%) and ampicillin (12.0%) as well as last-resort polymyxin B (31.7%), colistin (24.1%) and tigecycline (4.1%). The findings highlight potential prevalence of groundwater-borne antibiotic-resistant pathogenic *Acinetobacter* in the livestock environment.

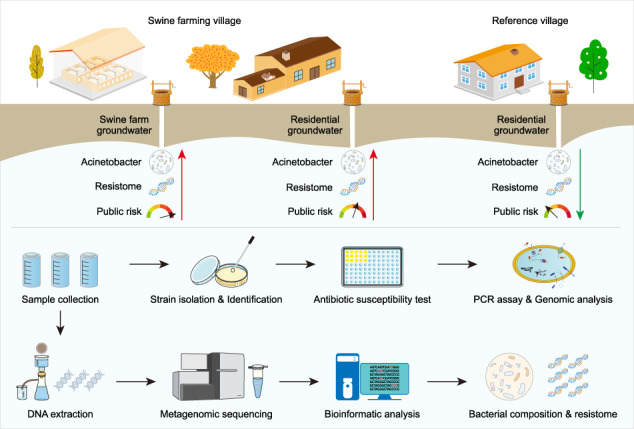

## Introduction

The massive use of antibiotics has exacerbated antimicrobial resistance (AMR) prevalence in the environment [1]. It is estimated that antibiotic-resistant bacteria (ARB) caused 1.27 million deaths in 2019, and the mortality is predicted to reach 10 million by 2050 [1, 2]. Given this great threat, AMR has been designated as an urgent public health threat by World Health Organization (WHO) [3], and is highlighted as one of the top six emerging environmental issues by United Nations Environment Programme [4, 5]. Livestock environments are hotspots for AMR development, transmission and prevalence [6]. ARB and antibiotic resistance genes (ARGs) have been detected prevalent in animal wastes, waste management systems [7–9] and the farm affected environments including water [10], agricultural soil [7], atmosphere [11] and food chains [12]. Under the “One-Health” concept, they will pose unpredictable risks to the public health [13].

AMR has become a growing concern in groundwater systems [14]. In rural China, groundwater is generally used for animal breeding processes. However, seepage of animal wastes will contaminate the surrounding groundwater, leading to the development of ARB and ARGs [15]. In northern China, carbapenem-resistant *Enterobacteriaceae* can be isolated from 5.3% animal farm groundwater [16]. High levels of ARGs are detected in aquaculture farm groundwater of central China [17]. Meanwhile, ARGs are prevalent in the swine farm groundwater of southern China [10]. These researches underline potential public health threats of groundwater-borne AMR, since groundwater acts as a transmission route of AMR from the environment to human body through drinking and daily use [14].

Our previous study indicates that *Acinetobacter* can be predominant in the swine farm groundwater with a positive correlation to the abundance of ARGs [10]. In clinical environments, these Gram-negative, non-fermented, rod-shaped bacteria are well-known opportunistic pathogens for their capabilities of escaping antibiotic biocidal actions and mutually representing new paradigms in pathogenesis, transmission, and resistance [18–21]. The isolates in clinical settings are reported resistant to most commonly used antibiotics, thus have become formidable obstacles in treating infectious diseases [22]. To minimize the public health threats, WHO recommends a routine detection of *Acinetobacter* in drinking water sources [23], for which antibiotic susceptibility tests have been standardized for this genus by Clinical and Laboratory Standards Institute (CLSI) [24]. Animal guts are habitats and environmental sources of antibiotic-resistant *Acinetobacter* [7, 9, 25]. Carbapenem-resistant *A*. *baumannii* and *A*. *calcoaceticus* as well as tigecycline-resistant *A*. *towneri* can be isolated from swine feces [26, 27]. Imipenem- and meropenem-resistant *A*. *junii* have been detected in the farm soil [28]. The *Acinetobacter* strains resistant to multiple first-line and newly approved antibiotics have been isolated from poultry manure, wastewater and soil in the poultry farming regions of China [29]. These studies highlight AMR risks of *Acinetobacter* in the livestock environment. However, groundwater is currently a blind spot of *Acinetobacter*-associated researches. Few studies have yet involved their abundance in non-farm groundwater [30, 31], and none analyzed their composition, resistance and pathogenicity. This limit further assessing environmental risks in the groundwater transmission route.

This study aims to elucidate the antibiotic resistance of *Acinetobacter* in the groundwater of the swine farming village using metagenomic and culture-dependent approaches, in comparison to the nearby non-farming village. Metagenomic assembling methods were used to profile the taxonomic composition and (acquired) antibiotic resistome of *Acinetobacter* in the groundwater. Metagenomic binning methods were used to analyze the antibiotic resistance and virulence potentials of *Acinetobacter* community, coupled with their genomic abundance in the groundwater. “High-risk” species were highlighted based on their antibiotic resistance, virulence and abundance. Meanwhile, *Acinetobacter* strains were isolated from the groundwater of the swine farming village. Their resistance profiles to 13 antibiotics were subsequently tested following the CLSI guideline. For polymyxins resistance, PCR assays were implemented to detect the associated genes, and whole genomes of highly resistant strains were sequenced to analyze potential mechanisms. The findings will expand knowledges of antibiotic-resistant *Acinetobacter* in the livestock environment.

## Materials and methods

### Study area and sample collection

In this study, a swine farming village (JZ) and a nearby (3.5 km) non-farming village (WT) in Guangxi of southern China were selected as the study area. In this distance, WT is considered having similar geographic and sanitary conditions with JZ, but limited influence by swine farming activities. JZ had 200 conventional semi-confined swine farms with a production of 50,000 heads/year, while WT only had a few animals for self-sufficient use. The swine farms lacked advanced waste treatment systems. Swine wastes were cleaned by farmers manually, and deposited in storage tanks. Then, the wastes were used for agricultural fertilization or transported to treatment plants for centralized treatment. To this end, the water systems, soil and air had been contaminated by farming activities [7, 10, 32, 33].

In December of 2018, 22 groundwater samples (average 6-m depth, the only water source in both villages) were collected from 3 well water groups: 13 wells in JZ residential houses (JZG), 5 wells in JZ swine farms (JZPG) and 4 wells in WT residential houses (WTG, Supplementary Fig. S1 of Supporting Information 1). It is noted that there were 200 farms in the village, therefore JZG were also very close to the farms despite located in residential houses. The groundwater in both JZG and JZPG were influenced by farming activities with antibiotic and ammonia contaminations [10]. Meanwhile, WT is much smaller than JZ, and 4 wells can represent the groundwater in the village. The water samples were collected in 10L sterilized plastic bottles, and then transported to the laboratory on ice. Basic water quality and antibiotic concentrations are listed in Supplementary Table S1 of Supporting Information 2.

### DNA extraction and metagenomic sequencing

Groundwater samples were filtered with sterilized membranes (0.22-μm pore size, 50-mm diameter). Total DNA on each membrane was extracted using DNeasy^®^ PowerSoil^®^ kit (QIAGEN, Germany) according to the manufacturer’s instruments. DNA yields were measured using a Qubit 2.0 fluorometer (Thermo Fisher Scientific, USA). Water volume and DNA yield of each sample are listed in Supplementary Table S2 of Supporting Information 2. Before sequencing, DNA was fragmented into 300 bp using an ultrasound machine (Covaris M220, USA), and then pair-end DNA library was conducted using TruSeq™ DNA Sample Prep Kit (Illumina, USA). Collectively, twenty-one groundwater samples (one WTG sample was not sequenced because of low DNA yields) were metagenomically sequenced on a Novaseq 6000 platform (Illumina, USA) by Majorbio Bio-Pharm Technology (Shanghai, China) with the sequence production of over 12 Gb/sample.

### Reads-based profiling of bacterial community and antibiotic resistome in the groundwater

Raw sequencing reads were quality-controlled and trimmed using KneadData v0.7.4 (https://huttenhower.sph.harvard.edu/kneaddata/) with default parameters. About 37.7–52.5 million clean reads were obtained for each sample (Table S2 of Supporting Information 2). The antibiotic resistome in the groundwater was analyzed using SARGs-OAP v2.0 (set: -l 25 -d 80 -e 1e-10) with the Structured Antibiotic Resistance Genes database [34]. Bacterial community composition was obtained using kraken2 classifier with the Genome Taxonomy Database (GTDB v202) [35, 36]. For each taxon, the maximum relative abundance lower than 0.001 were regarded as not detected.

### Assembly-based analysis of ARGs’ hosts

Metagenomic reads in all samples were merged and assembled to contiguous sequences (contigs) using Megahit v1.2.9 (default parameters) [37]. Open reading frames (ORFs) on the contigs were predicted using Prodigal v2.6.3 (set: -c -p meta) [38]. The ORFs were then clustered and dereplicated using CD-HIT v4.8.1 (set: -aS 0.9 -c 0.95) [39]. Salmon v0.13.1 was used to estimate and normalize ORFs’ abundance into the transcripts per million reads (TPM) unit [40]. Potential ARGs were searched against the SARGFam database using hmmscan v3.3.2 (set: --cut_ga -noali) [34]. The taxonomy of ARG-ORFs was determined using kraken2 with the GTDB database.

### Assembly-based analysis of the antibiotic resistome in *Acinetobacter*

The gene set of *Acinetobacter* was obtained by annotating ORFs’ taxonomy against the GTDB database using DIAMOND blastp v0.9.22 (set: --very-sensitive --id 90 --query-cover 90) [41]. Potential ARGs in the gene set were searched using RGI v5.1.1 (perfect and strict hits) with the Comprehensive Antibiotic Resistance Database (homolog model, v3.1.3) [42]. Potential acquired ARGs were searched using ResFinder v4.0 (set: -acq -t 0.8 -l 0.8) [43]. The taxonomy of ARG-contigs (a contig carrying at least one acquired ARG) were determined using kraken2 with the GTDB database. Functional genes and mobile gene elements (MGEs) in the contigs were searched against the NCBI nr database (downloaded on 2021.11.24) using DIAMOND blastp (set: --very-sensitive --id 80 --query-cover 80).

### Metagenomic binning analysis

Metagenomic assembled genomes (MAGs) were generated from each sample by Metabat2, Maxbin2 and CONCOCT assemblers in MetaWRAP pipelines v1.3 [44]. The output MAGs were filtered by bin_refinement module (set: -c 70 -x 5). The bacterial or archaeal genomic taxonomy was classified by GTDB-Tk v1.4.1 with universal marker genes [36]. Their genomic information including assembly quality, completion and contamination are provided in Table S3 of Supporting Information 2. Potential ARGs in each MAG were searched using DeepARG, a deep learning-based approach for ARG annotation (set: --model LS --model-version v2 --arg-alignment-overlap 0.8 --type prot --arg-alignment-identity 80 --arg-alignment-evalue 1e-10 --arg-num-alignments-per-entry 1000) [45]. Potential VFGs and MGEs were annotated against the Virulence Factor Database (VFDB, downloaded on 2021.02.05) [46] and the MGEs90 database (https://bench.cs.vt.edu/ftp/data/databases/) using DIAMOND blastp (set: --very-sensitive --id 80 --query-cover 80). It is considered that one VF exists when at least one VFG is detected in the genomes. The genomic coverage (as relative abundance) of each MAG was estimated using Bowtie2 and SAMtools [47, 48], and normalized by the genomic size (Mbp) and sequencing file size (Gb).

### Strain isolation and identification

Metagenomic analysis indicated that *Acinetobacter* was much more prevalent in the groundwater of JZ than that in WT (see result section), therefore culture-based methods were subsequently conducted for testing the antibiotic resistance of *Acinetobacter* in JZ groundwater. The *Acinetobacter* strains were isolated from 15 JZ groundwater samples (no colonies grew in the other three samples) using the Leeds Acinetobacter Medium (LAM) agar plate [49]. The purified isolates were then confirmed by Gram’s stain (negative), catalase (positive), nitrate reductase (negative) and oxidase tests (negative, Supplementary Fig. S2 of Supporting Information 1). Their 16S rRNA gene sequences were amplified and sequenced using universal primers (27F/1492R) by the Beijing Genomics Institute (BGI, China). The taxonomy was classified by aligning sequences to the reported species using NCBI Blastn online tools (https://blast.ncbi.nlm.nih.gov/Blast.cgi).

### Antibiotic susceptibility tests

The broth microdilution method was used to test antibiotic susceptibility following the CLSI guidelines v2018. Briefly, the strains were inoculated (5 × 10^5^ cells/mL in triplicates) in the cation-adjusted Mueller-Hinton broth (a final volume of 200 μL in 96-well plates) with each of 12 antibiotics at the breakpoint concentrations (ampicillin: 32 μg/mL; meropenem: 8 μg/mL; colistin: 4 μg/mL; polymyxin B: 4 μg/mL; gentamicin: 16 μg/mL; tobramycin: 16 μg/mL; doxycycline: 16 μg/mL; tetracycline: 16 μg/mL; ciprofloxacin: 4 μg/mL; levofloxacin: 8 μg/mL; gatifloxacin: 8 μg/mL; and sulfamethoxazole: 76 μg/mL). Since over 24.0% of the strains were resistant to polymyxins (colistin and polymyxin B, see result section), the minimum inhibitory concentration (MIC) for the resistant strains were determined by the two-fold serial dilution method. In addition, MICs of tigecycline for all strains were tested at a dilution range of 0.25–4 μg/mL. The resistance breakpoint was delineated as 1.0 μg/mL according to the European Committee on Antimicrobial Susceptibility Testing [50], since CLSI lacks the criteria for this antibiotic. *Escherichia coli* ATCC 25922 was used as the quality-control strain.

### Detection of intrinsic polymyxins resistance genes using PCR and whole-genomic sequencing methods

Polymyxins resistance can be mediated by plasmid-borne *mcr* genes and chromosomally encoded by two-component systems *pmr*ABC and lipopolysaccharide (LPS) biosynthesis genes (Supplementary Fig. S3 of Supporting Information 1) [51]. In this study, seven intrinsic resistance genes including *pmr*ABC, *lpx*ACD and *lps*B were tested for the strains using PCR methods [52]. The primers and PCR programs are summarized in Supplementary Table S4 of Supporting Information 2. The *mcr* genes were not tested since they were not detected by metagenomic analysis. Meanwhile, the draft genomes of ten highly resistant strains were sequenced on the HiSeq X Ten platform (Illumina, USA) by Majorbio Bio-Pharm Technology (details shown in Supplementary Text S1 of Supporting Information 1). The LPS biosynthesis genes including *lpx*ACD and *lps*B were searched in the genomes using DIAMOND blastp (set: --very-sensitive --id 80 --query-cover 80) with the VFDB database.

### Data visualization

R v4.0.3 was used for data statistics and visualization. Bar, stack and box plots were presented by ggplot2 v3.3.6. Venn plots were generated using venn v2.5–6. Sankey plots were drawn using networkD3 v0.4. Phylogenetic trees for MAGs were generated by gtdbtk infer module in GTDB-Tk v1.4.1. Phylogenetic relationships (16S rRNA gene) of the isolated strains were analyzed using MegaX with muscle alignment kit, maximum likelihood method, 100 bootstrap and kimura 2-parameter model. The phylogenetic trees were drawn by iTOL (https://itol.embl.de/). Genetic environments were drawn using gggenes v0.4.1. Schematic plots and figure layout were conducted by Adobe Illustrator CC v2019.

## Results

### Antibiotic resistome and bacterial community in the groundwater

**Fig. 1:**
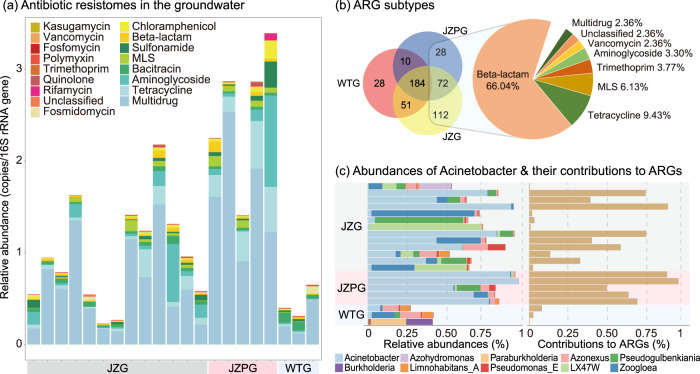
Antibiotic resistome and bacterial community composition in the groundwater. **a** Relative abundance of ARG types in the groundwater. **b** Unique and shared ARG subtypes in the groups. Values in the venn plot represent ARG numbers, and the percentages in pie plot are proportion of the ARGs only detected in JZG and JZPG. **c** Relative abundance of top ten genera with the *Acinetobacter* contribution to the resistome. ARG antibiotic resistance gene, MLS macrolide-lincosamide-streptogramin, JZG residential groundwater in the swine farming village, JZPG swine farm groundwater in the swine farming village, WTG residential groundwater in the reference village.

In this study, 485 ARGs against 16 antibiotic classes were detected in the groundwater using the metagenomic reads-based methods (Fig. 1a, Supplementary Table S5 of Supporting Information 2). The ARGs encoding multidrug resistance (average 64.6% of the resistome, hereafter), tetracyclines resistance (10.5%) and aminoglycosides resistance (9.9%) were prevalent in all samples. The abundance of the resistome in all JZPG (1.4–3.4 copies/16S rRNA gene) and some JZG (0.3–0.7 copies/16S rRNA gene) was much higher than that in WTG (0.3–0.7 copies/16S rRNA gene). Over 180 ARGs were shared by three groups, while 212 ARGs including 140 beta-lactam resistance genes were only detected in JZ groundwater (Fig. 1b). This suggests an increased abundance and diversity of the resistome in the farm affected groundwater.

In terms of microbial community, 63.6%-95.8% of metagenomic reads were classified to bacteria in the groundwater. Of the 158 detected genera (Supplementary Table S6 of Supporting Information 2), *LX47W* (maximum 71.1% of the bacteria in all samples, hereafter), *Zoogloea* (65.3%), *Pseudogulbenkiania* (55.6%), *Paraburkholderia* (22.4%), *Azohydromonas* (20.5%), *Azonexus* (15.8%), *Burkholderia* (16.8%) and *Limnohabitans_A* (6.5%) were detected in higher abundance (Fig. 1c). Comparatively, *Acinetobacter* was abundant in all JZPG (54.4–95.7%) and 8 JZG (~91.1%), while the abundance was much lower in WTG (0.2%–8.0%).

### Assembly-retrieved antibiotic resistome in *Acinetobacter*

**Fig. 2:**
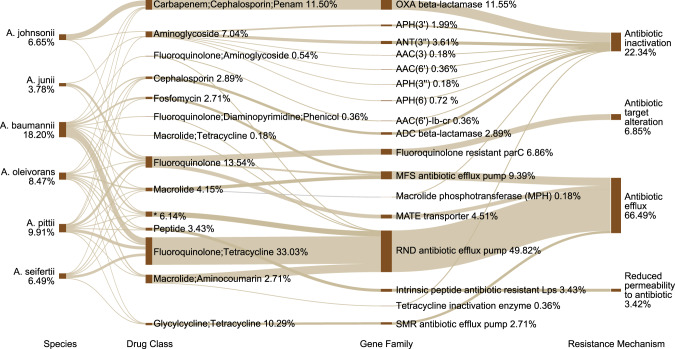
The antibiotic resistome in *Acinetobacter* community. The predominant six species are presented in the left. The percentages below species names are their proportions in *Acinetobacter* community. The right columns show the ARGs detected in these species, while the percentages are proportions of the ARGs in all *Acinetobacter* species. The complete results are summarized in Supplementary Table S7. Asterisk-marked antibiotic category includes macrolide, fluoroquinolone, lincosamide, carbapenem, cephalosporin, tetracycline, rifamycin, diaminopyrimidine, phenicol and penem.

A total of 99 ARGs against 23 antibiotic classes (multidrugs were separated into corresponding classes) were detected in *Acinetobacter* using the metagenomic assembling methods (Supplementary Table S7 of Supporting Information 2). They accounted for ~95.6% of total resistome in *Acinetobacter* dominant groundwater (Fig. 1c). The major hosts were *A*. *baumannii* (18.2% of *Acinetobacter* resistome, hereafter), *A*. *pittii* (9.9%), *A*. *johnsonii* (6.7%), *A*. *seifertii* (6.5%) and *A*. *junii* (3.8%) (Fig. 2). The predominant resistance mechanisms were antibiotic efflux (66.49%), antibiotic inactivation (22.34%) and antibiotic target alteration (6.85%). Tetracyclines resistance genes (50.18%) and fluoroquinolones resistance genes (53.61%) were the major ARG types (Fig. 2). Efflux pump genes associated with resistance-nodulation-cell division (RND, 49.82%), major facilitator superfamily pump (MFS, 9.39%) and multidrug and toxic compound extrusion transporter (MATE, 4.51%) were dominant gene families (Fig. 2). Meanwhile, OXA beta-lactamase (11.55%) and fluoroquinolone resistant parC (6.86%) families were also prevalent in *Acinetobacter*.

**Fig. 3:**
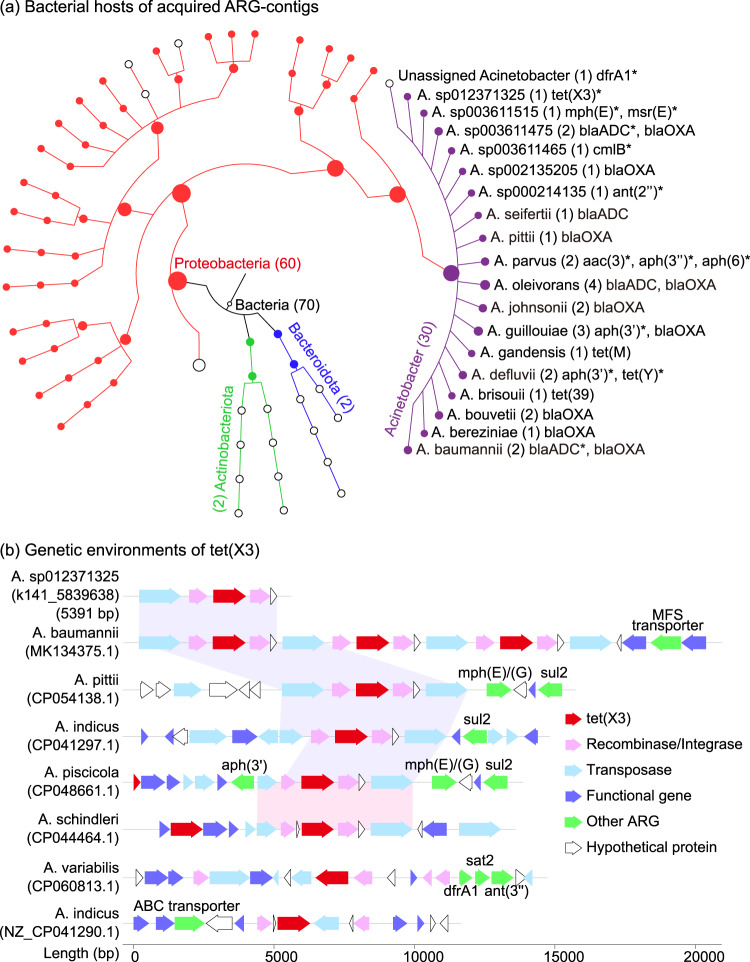
Profiles of acquired ARGs in the groundwater. **a** Potential hosts of the contigs in the groundwater. Values in brackets are contig numbers. Asterisk-marked ARGs mean that there were MGEs colocalized. **b** Genetic environments of *tet*(X3)-contig in the published *Acinetobacter* species. The accession number of each species is given in the bracket. Genes in a shadow have the same genetic environment.

A total of 70 contigs carrying acquired ARGs were detected in the groundwater (Fig. 3a, Supplementary Table S8 of Supporting Information 2). Therein, 30 contigs were classified to 18 *Acinetobacter* species. Two monooxygenase gene variants *tet*(X3) and *tet*(X6) conferring resistance to tigecycline were detected with higher abundance in *Acinetobacter* dominant groundwater (e.g., *tet*(X3) in JZPG5 and *tet*(X6) in JZPG4, Supplementary Table S8 of Supporting Information 2). In the NCBI nt database, the conservative genetic environment of *tet*(X3) has been reported in the plasmids of seven *Acinetobacter* species (Fig. 3b). Some ARGs conferring resistance to other antibiotic classes, such as *sul*2, *aph*(3’) and *mph*(E)/(G) were colocalized with this structure. Meanwhile, *tet*(X6) has been detected in the chromosomes and plasmids of six species including *A*. *baumannii*, *A*. *towneri*, *A*. *indicus*, *A*. *piscatorial*, *A*. *schindleri* and *A*. *pseudolwoffii*. Besides *tet*(X), trimethoprim resistance gene *dfr*A were also observed colocalized with multiple ARGs and MGEs in *Acinetobacter* and other bacteria (Supplementary Fig. S4 of Supporting Information 1). Meanwhile, several other ARGs including *mph*(E), *msr*(E), *bla*ADC, *cml*B, *ant*(2”), *aac*(3), *aph*(3), *aph*(6) and *tet*(Y) were also colocalized with MGEs.

### MAG-retrieved antibiotic resistance and pathogenicity in *Acinetobacter*

**Fig. 4:**
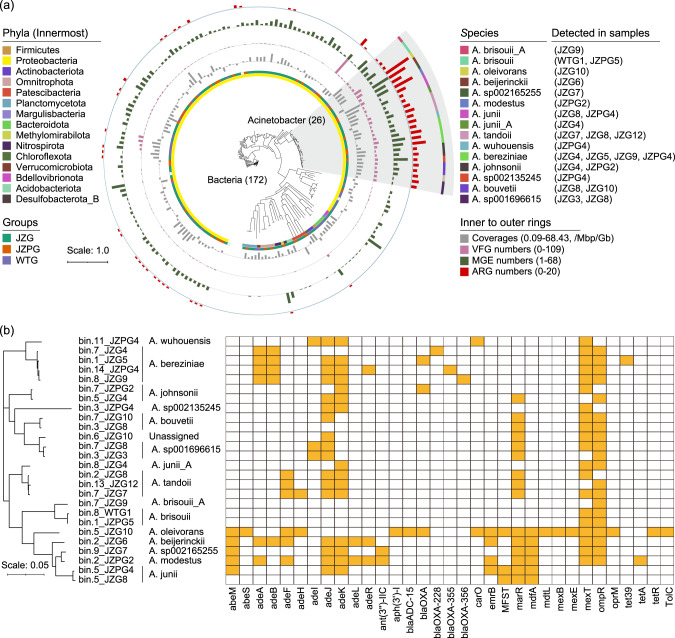
The genomic antibiotic resistance and pathogenicity of *Acinetobacter* in the groundwater. **a** The phylogenetic tree of the MAGs. Values in brackets are genome numbers of bacteria and *Acinetobacter*. *Acinetobacter* genomes are highlighted in the shadow. Rings represent phylum, group, genomic coverage as well as numbers of VFGs, MGEs and ARGs from the innermost to the outermost. **b** ARG compositions in *Acinetobacter* genomes. ARG antibiotic resistance gene, VFG virulence factor gene, MGE mobile gene element, JZG residential groundwater in the swine farming village, JZPG swine farm groundwater in the swine farming village, and WTG residential groundwater in the reference village.

A total of 172 bacterial MAGs and 8 archaeal MAGs were obtained in the groundwater (Supplementary Table S3 of Supporting Information 2). The bacterial MAGs were classified to 15 phyla, and therein 81.4% were classified to *Proteobacteria* (Fig. 4a). There were 26 MAGs classified to 15 *Acinetobacter* species. They were abundant (~68.4 copies/Mbp/Gb) in JZ groundwater, and carried more ARGs (a sum of 30 subtypes), MGEs and VFGs than other bacteria (Fig. 4a, Supplementary Table S9 of Supporting Information 2). Specifically, *A*. *oleivorans* carried 20 ARGs, which was more than the other *Acinetobacter* species (Fig. 4b). *A*. *modestus* carried 14 ARGs, and was dominant in JZPG2 sample (93.1% of metagenomic reads). *A*. *bereziniae* was detected in four groundwater samples, and carried eight multidrug efflux pump genes (*ade*ABJKR, *mex*T, *omp*R and *tet*39) and four beta-lactamase resistance genes (*bla*OXA, *bla*OXA-355, *bla*OXA-228 and *bla*OXA-356). Over 30 MGE classes involving recombinase, integrase and transposase were detected in *Acinetobacter* (Supplementary Table S9 of Supporting Information 2). These MGEs accounted for 0.1–1.9% of the genes in each MAG. The IS gene family was the most prevalent, and therein IS3 was detected in all species. Several ARGs including *aac*(6’)-I (found in *A*. *wuhouensis*), *omp*R (found in *A*. *brisouii*), and *ade*IJK (found in multiple species) were colocalized with MGEs.

**Fig. 5:**
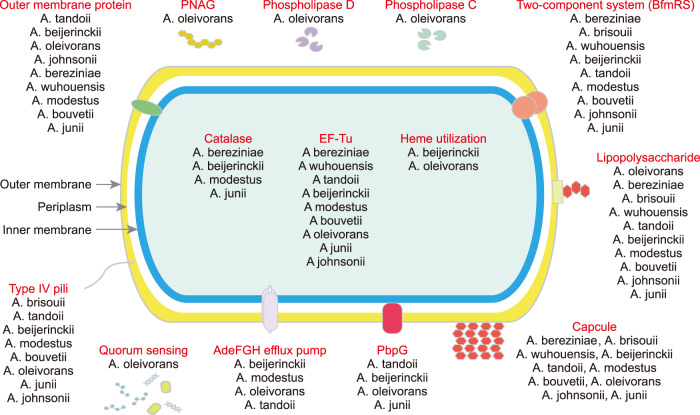
Potential virulence factors in *Acinetobacter* community.

In this study, 99 VFGs referring to 14 VF classes were detected in *Acinetobacte*r genomes (Fig. 5, Supplementary Table S9 of Supporting Information 2). The VFGs associated with capsule (ten species), LPS (ten species), two-component system BfmRS (nine species), EF-Tu (nine species) and outer membrane protein (nine species) were commonly detected, while PNAG, phospholipase C/D and quorum sensing were only found in *A*. *oleivorans*. In addition, ten VF classes including pbpG, adeFGH efflux pump, type IV pili, heme utilization, LPS, outer membrane protein, capsule, BfmRS, catalase and EF-Tu were detected in *A*. *beijerinckii*. The latter six were also detected in *A*. *bereziniae*. Collectively, *A*. *oleivorans*, *A*. *bereziniae*, *A*. *modestus* and *A*. *beijerinckii* might pose potential health risks since they were abundant (>1copy/Mbp/Gb) in the groundwater, and carried multiple (≥10 subtypes) ARGs and VFGs.

### Antibiotic susceptibility of the isolated strains

**Fig. 6:**
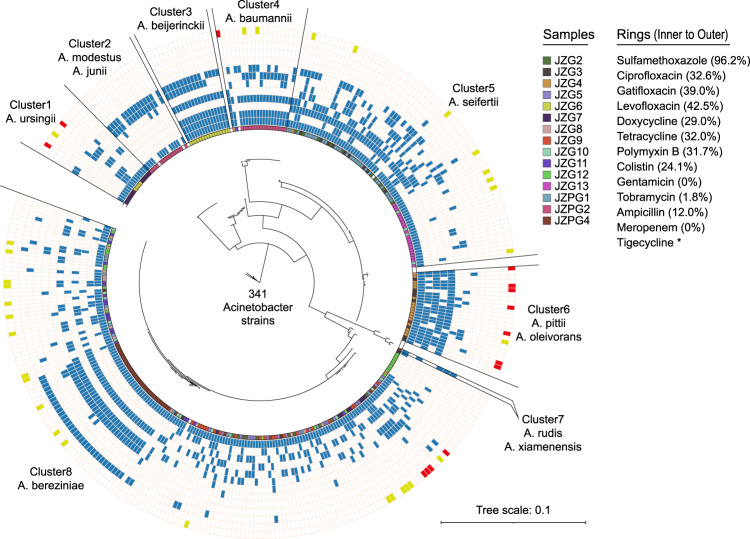
The phylogenetic relationship of *Acinetobacter* strains with the antibiotic resistance phenotype. The strains are clustered into eight groups using 16S rRNA gene sequences with 99% similarity and 95% coverage. Rings represent samples as well as resistance profiles to sulfamethoxazole, ciprofloxacin, gatifloxacin, levofloxacin, doxycycline, tetracycline, polymyxin B, colistin, gentamicin, tobramycin, ampicillin, meropenem and tigecycline from the innermost to the outermost. Percentages in brackets are resistance ratios. Blue represents resistance, while yellow means MIC = 0.5 μg/mL of tigecycline, and red means MIC = 1.0 μg/mL of tigecycline.

In this study, 341 strains were isolated from the groundwater. They were classified to 11 species including *A*. *ursingii*, *A*. *modestus*/*junii* (strains might be either of them since their 16S rRNA gene sequences were very similar (> 99.0%), hereafter), *A*. *beijerinckii*, *A*. *baumannii*, *A*. *seifertii*, *A*. *pittii*/*oleivorans*, *A*. *rudis*/*xiamenensis*, and *A*. *bereziniae*. Their resistance profiles to 13 antibiotics were presented in Fig. 6. Detailly, over 80.0% of the strains were resistant to at least two antibiotics, and nearly 70.0% were multidrug-resistant (≥three antibiotics). These multidrug-resistant strains were mainly classified to *A*. *beijerinckii*, *A*. *pittii*/*oleivorans*, *A*. *baumannii*, *A*. *seifertii* and *A*. *bereziniae*. Among all strains, the resistance ratios were higher to sulfamethoxazole (resistance ratio: 96.2%, covering all species), fluoroquinolones including levofloxacin (42.5%, none of *A*. *ursingii*, *A*. *modestus*/*junii* and *A*. *beijerinckii*), gatifloxacin (39.0%, none of *A*. *modestus*/*junii* and only one *A*. *baumannii*) and ciprofloxacin (32.6%, none of *A*. *modestus*/*junii*), as well as tetracyclines including tetracycline (32.0%, none of *A*. *ursingii* and *A*. *beijerinckii*) and doxycycline (29.0%, none of *A*. *modestus*/*junii* and only one *A*. *baumannii*). A few strains were resistant to ampicillin (12.0%, all classified to *A*. *bereziniae*) and tobramycin (1.8%, *A*. *seifertii* and *A*. *bereziniae*). However, none strains were resistant to gentamicin and meropenem. The MICs of tigecycline for most strains (87.1%) were lower than 0.25 μg/mL, while 14 strains including two *A*. *ursingii*, one *A*. *baumannii*, seven *A*. *pittii*/*oleivorans* and four *A*. *bereziniae* were tigecycline-resistant (MIC = 1 μg/mL).

Over 24.0% of the strains were resistant to colistin or polymyxin B (Fig. 6). Notably, thirty-one strains were resistant to both antibiotics (MICs ≥16 μg/mL, Supplementary Fig. S5 of Supporting Information 1). PCR results showed that the detection ratios of seven intrinsic resistance genes were inconsistent across the strains (Supplementary Fig. S6 of Supporting Information 2). Meanwhile, genomic analysis indicated that the amino acid (aa) sequences of *lps*B and *lpx*ACD in ten resistant strains significantly varied to the reference genome (Supplementary Table S10 of Supporting Information 2). Detailly, *lps*B with 13 aa mismatches was only detected in three genomes by PCR and whole-genome sequencing. *Lpx*A with 50 aa mismatches was detected in all genomes, but not detected by the PCR method. Similarly, *lpx*C was detected in all genomes with 28 aa mismatches, but detected in only four strains using the PCR method. However, *lpx*D was detected in two genomes by the PCR method, but not detected in the draft genomes. In addition, the sequences of *lpx*D in three genomes were greatly distinct (59 aa mismatches) to the reference genome.

## Discussion

This study detected multiple ARGs and *Acinetobacter* species in the swine farm groundwater and the affected residential groundwater. Their abundance was much higher in the groundwater of the farming village than that of the nearby non-farming village, implying the influence of farming activities to both bacterial community and antibiotic resistome. The composition of *Acinetobacter* species was spatially heterogenous in the groundwater, and several non-*A*. *baumannii* species including *A*. *oleivorans*, *A*. *bereziniae*, *A*. *modestus* and *A*. *beijerinckii* were prevalent in farm-affected groundwater. Generally, *Acinetobacter* is ubiquitous existence in natural environments including soil, fresh water, ocean and sediment, as well as animal, plant and human body [53, 54]. However, its abundance may be higher in human- (urban sewage and hospitals) or animal-contaminated (livestock wastewater) environments [9, 25, 31], in where *Acinetobacter* is commonly associated with AMR prevalence, and as an important vector of VFs causes an increased risk to circulatory and respiratory systems [55]. Besides environmental factors, several cellular traits contribute to its prevalence in the environment, including 1) bacterial capsules and extracellular substances against environmental stress [55, 56]; 2) quorum sensing and biofilm formation for population density maintenance [57]; 3) micronutrient acquisition systems for acquiring resources in oligotrophic environments [58]; and 4) bacterial toxins directly killing other bacteria [59]. These traits not only endow *Acinetobacter* with robust competitive advantages for resources, but are also associated with pathogenicity to human and intrinsic resistance to antimicrobial agents [60]. Collectively, *Acinetobacter* might threaten the local public health via the transmission route of drinking the groundwater.

In this study, the antibiotic resistance varied across the *Acinetobacter* species. For example, all ampicillin-resistant strains were classified to *A*. *bereziniae*; and all *A*. *modestus*/*junii* strains were susceptible to fluoroquinolones. The isolated strains were widely resistant to several antibiotic classes including sulfonamides, quinolones, tetracyclines and polymyxins, but was more susceptible to meropenem, ampicillin, gentamicin and tobramycin. Comparatively, the China Antimicrobial Resistance Surveillance System (http://www.carss.cn/, 2021) reported that over half of the clinic-relevant *Acinetobacter* strains were resistant to imipenem (resistance ratio: 65.6%), meropenem (66.5%), levofloxacin (56.0%), ciprofloxacin (66.5%), gentamicin (62.3%) and ampicillin-sulbactam (59.1%), but they were more susceptible to colistin (1.6%), polymyxin B (0.7%) and tigecycline (2.5%). These adverse results indicated a different AMR development process between the livestock and clinical environment.

The antibiotic resistome in *Acinetobacter* are currently limited studied as a whole. A recent study summarized ARG contents in 21 non-*A*. *baumannii* species, and elucidated that beta-lactams and aminoglycosides resistance genes as well as the efflux pump resistance mechanisms are commonly detected in *Acinetobacter* [61]. The beta-lactams resistance, especially last-resort carbapenems, has obtained growing concerns in livestock-associated *Acinetobacter* researches [62]. Imipenem-resistant *A. baumannii*, carbapenem-resistant *A*. *calcoaceticus*, imipenem- and meropenem-resistant *A*. *junii* have been isolated from animal feces and farm-affected soil [26–28], suggesting that livestock environments are potential hotspots for carbapenem-resistant *Acinetobacter* prevalence. In terms of aminoglycosides resistance, *aph*(6), *ant*(3”), *aph*(3”) and *str*A are the most prevalent ARGs in *Acinetobacter* [61]. The former three are detected in *A*. *pittii*, *A*. *indicus* and *A*. *haemolyticus*, while the latter one is carried by *A*. *pittii*, *A*. *nosocomialis*, *A*. *radioresistens*, *A*. *seifertii*, *A*. *haemolyticus*, *A*. *towneri*, *A*. *johnsonii* and *A*. *ursingii*. However, despite beta-lactams and aminoglycosides resistance genes were detected using the metagenomic methods, the isolated strains in this study were rarely resistant to meropenem and aminoglycosides, implying weak activities of the relevant ARGs. Four efflux pump families were detected in multiple *Acinetobacter* species. The impressive genetic plasticity of the genomes can enhance the intrinsic resistance attributable to these efflux pumps or introduce new resistance by rapid gene mutation, recombination and integration [22, 63]. Meanwhile, *Acinetobacter* can acquire antibiotic resistance through obtaining plasmid-borne ARGs colocalized with MGEs [64]. These might contribute to the multidrug resistance in *Acinetobacter* of this study.

A growing amount of polymyxins-resistant *Acinetobacter* strains have been isolated from different regions of the world [65]. Although polymyxins have yet been used in animal breeding since 2016, the resistance ratio in this study was at a relatively higher level in comparison to the reported environments (resistant ratios: 0.2–53.1%) [65], implying that other environmental or cellular factors might influence the resistance. The polymyxins resistance in *Acinetobacter* can be mediated by both acquired and intrinsic resistance mechanisms [51]. Since the acquired resistance genes (the *mcr* family) were not detected using the metagenomic methods, the intrinsic mechanisms might contribute to the resistance. The processes involve 1) adding phosphoethanolamine (PetN) to lipid A; 2) mutations of lipid A biosynthesis genes leading to its complete loss; 3) low expression of proteins for outer membrane stability; and 4) deficient expression of LPS biosynthesis cofactors [51]. Diverse detection of the key genes in these processes suggested that the polymyxin resistance might be increased by gene mutation, recombination and insertion through influencing LPS structures and biosynthesis [66].

With the global spread of carbapenems and polymyxins resistance genes, tigecycline has been raised to be another last-line regimen for treating a vast of clinical infections caused by multidrug-resistant bacteria [67]. However, tigecycline resistance introduced by *tet*(X) inhibits clinical effectiveness of tigecycline [67]. In the GeneBank database, three *tet*(X) variants including *tet*(X3), *tet*(X5) and *tet*(X6) are observed in several *Acinetobacter* species, which are all associated with livestock environments [27]. They are generally colocalized with several MGEs referring to insertion, transposon and integron, and thereby can be transferred across species [27]. In the livestock environment, tigecycline-resistant *Acinetobacter* strains carrying *tet*(X) have been isolated from animal feces, wastewater and farm-affected soil [27], but have yet to be reported in farm affected groundwater. This study highlights the needs of future larger-scale investigation on tigecycline-resistant *Acinetobacter* in the livestock groundwater. However, the breakpoint value of tigecycline for *Acinetobacter* has not been standardized by the international communities: CLSI lacks the criteria for this antibiotic, and the breakpoint value is delineated as 1.0 μg/mL [50], 2.0 μg/mL [68] and 8.0 μg/mL [29] in different standards. Therefore, standardizing the global criteria is urgent for surveilling and controlling tigecycline-resistant *Acinetobacter* in the environment.

One of the most arresting features of *Acinetobacter* is their powerful capability to cause infectious diseases [69]. Consensus supports that multi-factorial and combinatorial strategies with at least 16 gene islands are associated with virulence [70]. Among these VFs, quorum sensing, biofilm formation and efflux pumps are associated with self-protection and population density regulation when facing environmental stress [70]. LPS, capsule, outer membrane proteins and phospholipase are associated with cellular virulence [70]. Complex pathogenicity traits also confer them competitive advantages and anti-stress abilities [71]. In the clinical environment, *A*. *baumannii* is one of the most notorious pathogens for their high rates of pathogenicity and antibiotic resistance [2]. However, this study detected multiple VFGs relating to 11 virulence factors in non-*A*. *baumannii* species, such as *A*. *oleivorans*, *A*. *beijerinckii*, *A*. *seifertii*, *A*. *bereziniae* and *A*. *modestus*. This implies that these species might also pose public health risks in the livestock environment.

Conclusively, this study reported notable prevalence of *Acinetobacter* with severe antibiotic resistance in the swine farm and nearby residential groundwater. Compared with *A*. *baumannii* in the clinical environment, more species in the groundwater deserve our concerns because of their prevalence, antibiotic resistance and pathogenicity. Complex intrinsic and acquired mechanisms conferred *Acinetobacter* resistant to multiple first-line and last-resort antibiotics. Additionally, diverse VFs might endow them with invasive abilities to human body and competitive advantages in groundwater ecosystems. Future studies are suggested to investigate the antibiotic resistance of groundwater-borne *Acinetobacter* at a larger geographical scale, and to assess the public health risks arising from multidrug-resistant pathogenic *Acinetobacter* using the “One Health” methods.

## Supplementary information

Supplementary Information

Supplementary Table

## Data Availability

The metagenomic and draft genomic data in this study are available in the National Microbiology Data Center (http://nmdc.cn/) with the project accession number of NMDC10017956.
