# Supplementary material for: Swine farm groundwater is a hidden hotspot for antibiotic-resistant pathogenic Acinetobacter
Source: ISME Commun. 2023 Apr 20;3:34. doi: 10.1038/s43705-023-00240-w (PMC10119254; doi:10.1038/s43705-023-00240-w)
Supplement: Supplementary file 1 — Supplementary Information [file 43705_2023_240_MOESM1_ESM.docx]

**Swine farm groundwater is a hidden hotspot for antibiotic-resistant pathogenic *Acinetobacter***

Fang-Zhou Gao ^1,2^, Liang-Ying He ^1,2^, Xin Chen ^1^, Jing-Liang Chen ^1^, Xinzhu Yi ^3^, Lu-Xi He ^1,2^, Xin-Yi Huang ^1,4^, Zi-Yin Chen ^1,2^, Hong Bai ^1,2^, Min Zhang ^1,2^, You-Sheng Liu ^1,2^, Guang-Guo Ying ^1,2^

^1^ SCNU Environmental Research Institute, Guangdong Provincial Key Laboratory of Chemical Pollution and Environmental Safety & MOE Key Laboratory of Theoretical Chemistry of Environment, South China Normal University, Guangzhou 510006, P. R. China

^2^ School of Environment, South China Normal University, University Town, Guangzhou 510006, P. R. China

^3^ Institute of Ecological Science, Guangzhou Key Laboratory of Subtropical Biodiversity and Biomonitoring, Guangdong Provincial Key Laboratory of Biotechnology for Plant Development, School of Life Sciences, South China Normal University, Guangzhou 510631, P. R. China

^4^ MRC-University of Glasgow Centre for Virus Research, 464 Bearsden Road, Glasgow, G61 1QH, UK

Corresponding authors’ e-mail:

liangying.he@m.scnu.edu.cn (Liang-Ying He)

guangguo.ying@m.scnu.edu.cn (Guang-Guo Ying)

This file contains: Text S1 with the attached table and Figure S1-6.

**Text S1. Genomic sequencing and assembling for polymyxins-resistant strains.**

Genomic DNA of ten polymyxins-resistant strains was extracted using Wizard^®^ Genomic DNA Purification Kit (Promega, China) according to the manufacturer’s instruments. The purified DNA was quantified by a TBS-380 fluorometer (Turner BioSystems, USA) and sequenced (2 × 150 bp, sequencing depths > 300-fold genomes) on the Illumina HiSeq X Ten platform by Majorbio Bio-Pharm Technology. Raw reads were quality-controlled and trimmed by KneadData v0.7.4 pipeline. Genomic assembling was implemented using SOAPdenovo2 [1]. After that, Glimmer was used for gene prediction [2]; tRNA-scan-SE was used for tRNA prediction [3]; and Barrnap (github.com/tseemann/barrnap) was used for rRNA prediction. The above steps were implemented using default parameters. The genomic quality was listed in the following table:

**Attached table of Text S1.** The genomic quality of ten polymyxin-resistant strains.

| Genomes | Clean  Reads | Sequencing  Depth | Genome  size (Mbp) | Scaffold  No. | Largest Scaffold  Length (bp) | N50 (bp) | G+C (%) | Gene  No. | tRNA  No. | rRNA  No. |
| --- | --- | --- | --- | --- | --- | --- | --- | --- | --- | --- |
| JZG2_16 | 4261328*2 | 336.86 | 3.92 | 53 | 656426 | 292264 | 38.493 | 3698 | 72 | 3 |
| JZG2_10 | 4044608*2 | 310.31 | 4.03 | 145 | 533014 | 356218 | 38.518 | 3793 | 69 | 3 |
| JZG2_13 | 4265422*2 | 336.01 | 3.93 | 58 | 657168 | 292244 | 38.482 | 3695 | 71 | 2 |
| JZG6_10 | 4636215*2 | 392.18 | 3.64 | 80 | 328997 | 188322 | 38.165 | 3420 | 72 | 2 |
| JZG6_11 | 4143058*2 | 352.65 | 3.65 | 99 | 329199 | 159535 | 38.157 | 3426 | 74 | 2 |
| JZPG2_3 | 3764118*2 | 326.82 | 3.63 | 90 | 296705 | 107175 | 38.295 | 3520 | 76 | 3 |
| JZPG2_5 | 4228950*2 | 364.54 | 3.63 | 85 | 296639 | 107144 | 38.285 | 3545 | 80 | 3 |
| JZPG2_4 | 3977002*2 | 352.1 | 3.62 | 86 | 295892 | 104764 | 38.301 | 3545 | 72 | 2 |
| JZPG2_7 | 3936970*2 | 342.55 | 3.62 | 85 | 295724 | 107159 | 38.288 | 3536 | 69 | 3 |
| JZPG2_6 | 3675457*2 | 320.58 | 3.62 | 83 | 295719 | 109416 | 38.289 | 3542 | 76 | 3 |


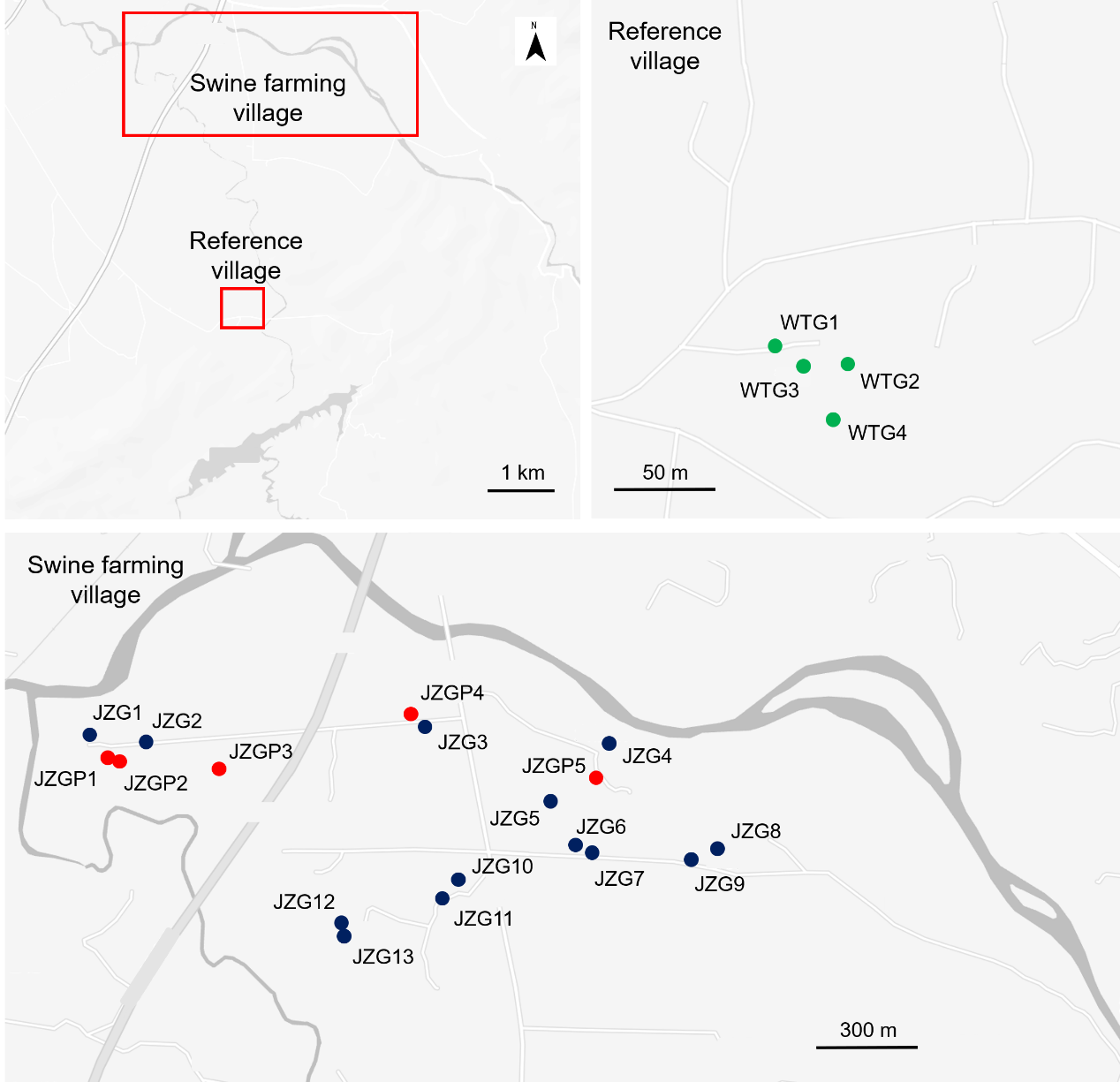


**Figure S1.** Sampling maps of this study. Green circles are well sites in the reference village; Dark blue circles are well sites of residential houses in swine farming village; and red circles are well sites of swine farms in swine farming village. The maps were downloaded from https://map.baidu.com/.


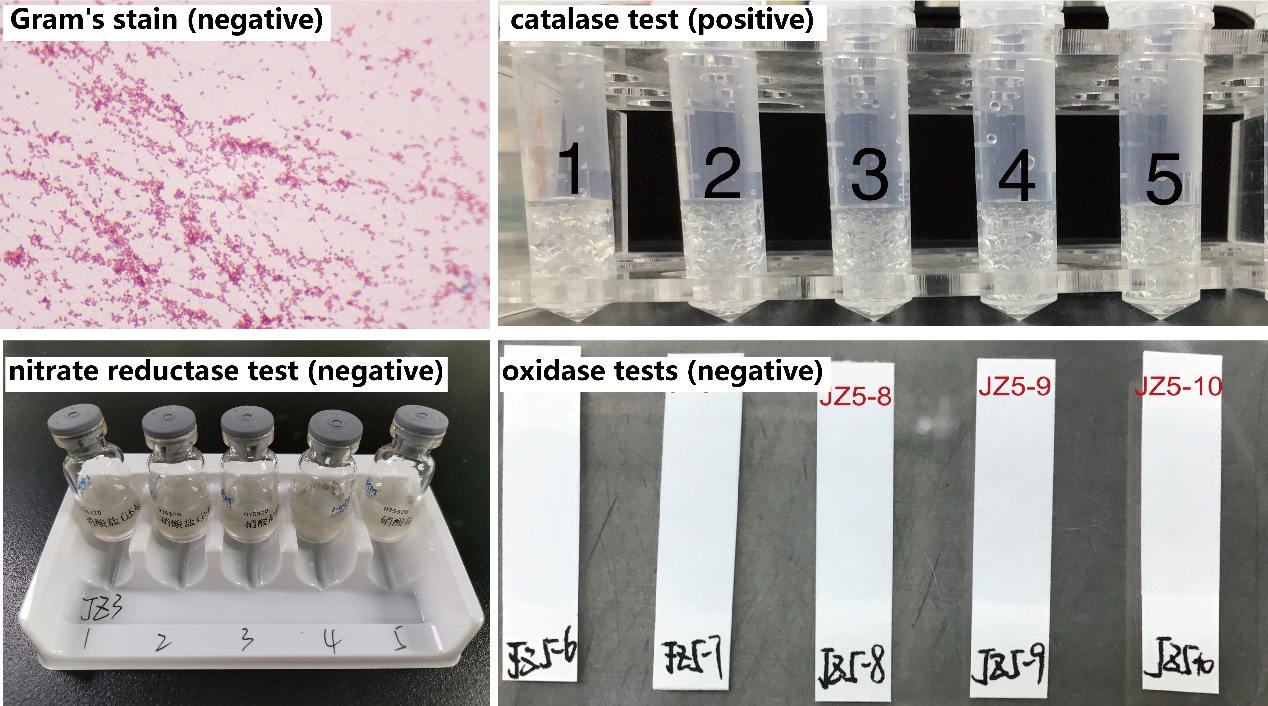


**Figure S2.** Examples of *Acinetobacter* biochemical identification.


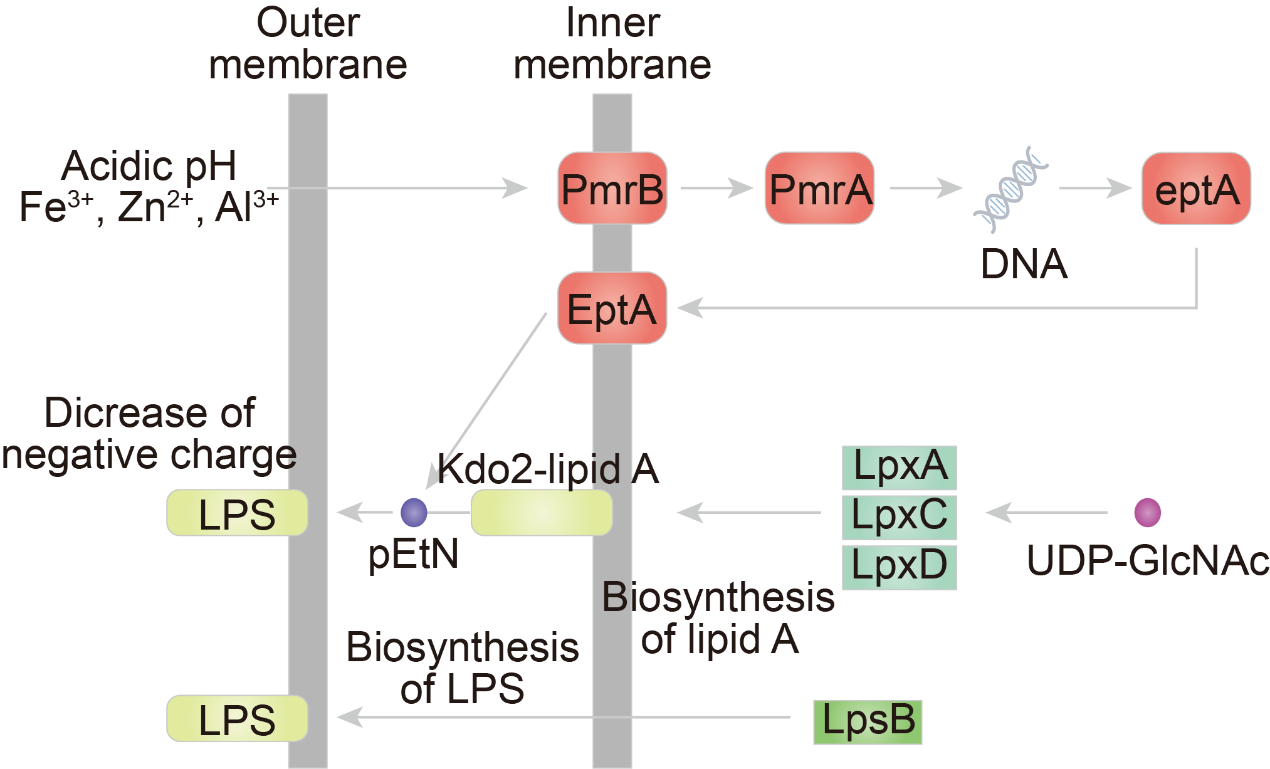


**Figure S3.** Polymyxins resistance mechanisms associated with lipopolysaccharide biosynthesis. Pathways were acquired from KEGG database (https://www.kegg.jp/).


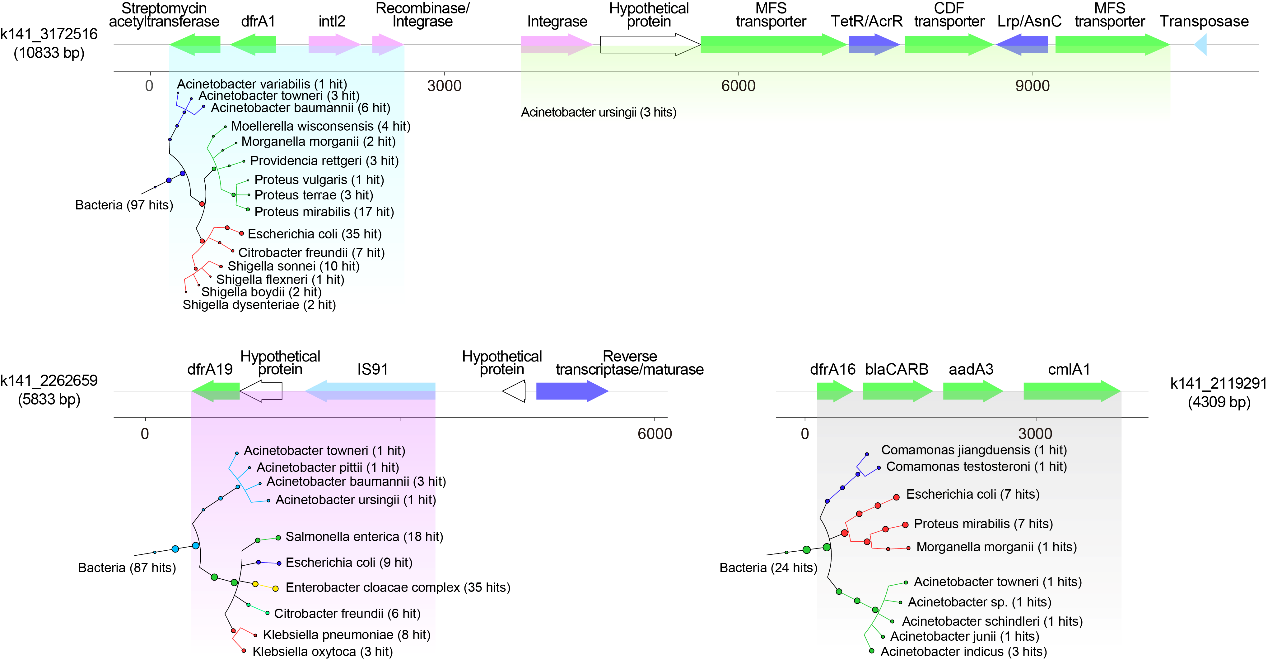


**Figure S4.** Genetic structures and hosts of *dfrA*-contigs.


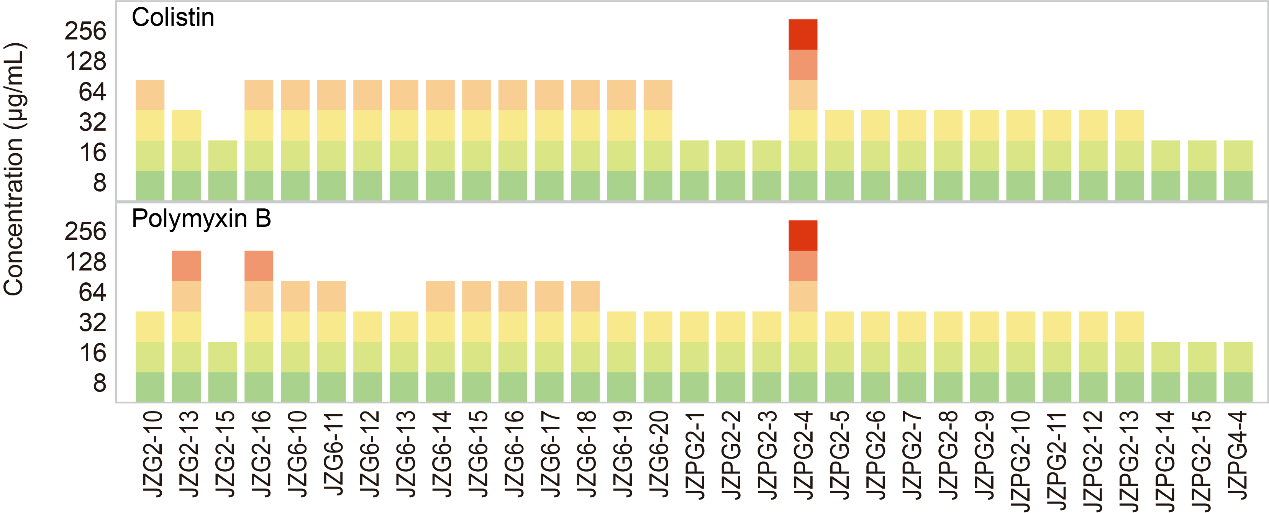


**Figure S5.** Polymyxins MICs for the resistant strains.


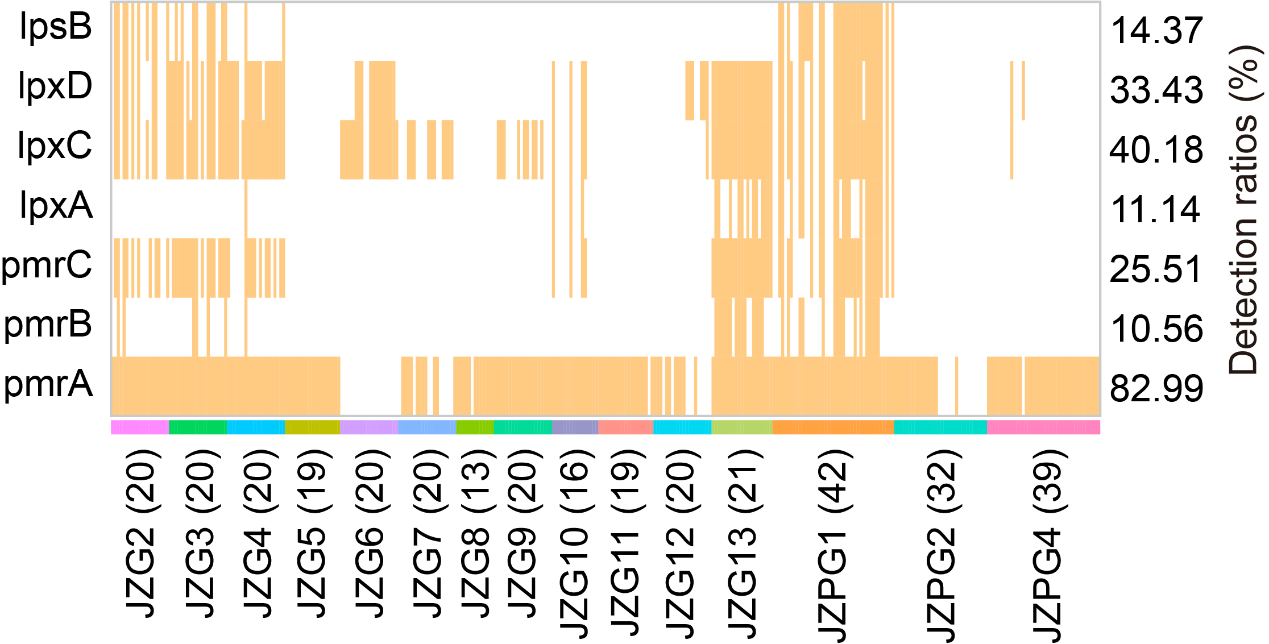


**Figure S6.** Detection ratios of seven intrinsic polymyxins resistance genes.

**References**

1. Luo, R, Liu, B, Xie, Y, Li, Z, Huang, W, Yuan, J, et al. SOAPdenovo2: an empirically improved memory-efficient short-read de novo assembler. Gigascience. 2012; 1:18.

2. Delcher, AL, Bratke, KA, Powers, EC, Salzberg, SL. Identifying bacterial genes and endosymbiont DNA with Glimmer. Bioinformatics. 2007; 23;673–679.

3. Torres, AG, Reina, O, Stephan-Otto Attolini, C, Ribas de Pouplana, L. Differential expression of human tRNA genes drives the abundance of tRNA-derived fragments. PNAS. 2019; 116:8451–8456.
